# Filamentous Pseudomonas Phage Pf4 in the Context of Therapy-Inducibility, Infectivity, Lysogenic Conversion, and Potential Application

**DOI:** 10.3390/v14061261

**Published:** 2022-06-10

**Authors:** Damir Gavric, Petar Knezevic

**Affiliations:** PK Lab, Department of Biology and Ecology, Faculty of Sciences, University of Novi Sad, Trg Dositeja Obradovica 2, 21000 Novi Sad, Vojvodina, Serbia; damir.gavric@dbe.uns.ac.rs

**Keywords:** *Pseudomonas aeruginosa*, Pseudomonas phage Pf4, virulence factors, biofilm, phage induction, therapy

## Abstract

More than 20% of all *Pseudomonas aeruginosa* are infected with Pf4-related filamentous phage and although their role in virulence of *P. aeruginosa* strain PAO1 is well documented, its properties related to therapy are not elucidated in detail. The aim of this study was to determine how phage and antibiotic therapy induce Pf4, whether the released virions can infect other strains and how the phage influences the phenotype of new hosts. The subinhibitory concentrations of ciprofloxacin and mitomycin C increased Pf4 production for more than 50% during the first and sixth hour of exposure, respectively, while mutants appearing after infection with obligatory lytic phage at low MOI produced Pf4 more than four times after 12–24 h of treatment. This indicates that production of Pf4 is enhanced during therapy with these agents. The released virions can infect new *P. aeruginosa* strains, as confirmed for models UCBPP-PA14 (PA14) and LESB58, existing both episomally and in a form of a prophage, as confirmed by PCR, RFLP, and sequencing. The differences in properties of Pf4-infected, and uninfected PA14 and LESB58 strains were obvious, as infection with Pf4 significantly decreased cell autoaggregation, pyoverdine, and pyocyanin production, while significantly increased swimming motility and biofilm production in both strains. In addition, in strain PA14, Pf4 increased cell surface hydrophobicity and small colony variants’ appearance, but also decreased twitching and swarming motility. This indicates that released Pf4 during therapy can infect new strains and cause lysogenic conversion. The infection with Pf4 increased LESB58 sensitivity to ciprofloxacin, gentamicin, ceftazidime, tetracycline, and streptomycin, and PA14 to ciprofloxacin and ceftazidime. Moreover, the Pf4-infected LESB58 was re-sensitized to ceftazidime and tetracycline, with changes from resistant to intermediate resistant and sensitive, respectively. The obtained results open a new field in phage therapy—treatment with selected filamentous phages in order to re-sensitize pathogenic bacteria to certain antibiotics. However, this approach should be considered with precautions, taking into account potential lysogenic conversion.

## 1. Introduction

*Pseudomonas aeruginosa* is an opportunistic pathogen known to cause various infections in humans, from mild, such as otitis externa, to life-threatening ones [1]. As a human pathogen, it most often colonizes the epithelium of the lungs and urinary tract [2]. Patients with burn wounds, AIDS, and cystic fibrosis (CF) are particularly at risk of developing serious *P. aeruginosa* infections, which account for a high death rate in this population [3,4,5]. Moreover, *P. aeruginosa* has the ability to colonize medical instruments and is therefore one of the most common nosocomial pathogens. 

This bacterium possesses a wide range of virulence factors that can be divided into those bound to the cell (e.g., lipopolysaccharides, flagella, pili) and those secreted from the cell (e.g., proteases, elastases, exotoxins, pyocyanin). However, the most important role in the pathogenicity of *P. aeruginosa* is its ability to form biofilms. After binding to a surface, *P. aeruginosa* undergoes a series of changes to adapt to a new way of life. Surface-bound cells grow and form microcolonies, while also producing an extracellular matrix. This extracellular matrix consists of exopolysaccharides, extracellular DNA (eDNA), and proteins, playing a role in biofilm adhesion and protection [6,7]. As the biofilm matures, the bacteria undergo physiological changes and become more resistant to environmental stresses. Due to its metabolic versatility, intrinsic and acquired antibiotic resistance, as well as biofilm formation and production of numerous virulence factors, *P. aeruginosa* represents a serious global health concern.

Much has been completed to isolate *P. aeruginosa* specific bacteriophages in order to use these viruses as alternative antimicrobial agents. The most studied bacteriophages are the tailed phages, and the reason behind this, is the ease of their isolation and multiplication. However, it is known that *P. aeruginosa* can also be infected by filamentous phages (Pf). This particularly interesting but insufficiently examined phage group, belonging to the order *Tubulavirales*, differs significantly from other bacteriophages in terms of morphology and life cycle [8]. Filamentous phages have virions in which the major coat protein is helically organized around circular, positive-sense, single-stranded DNA ((+) ssDNA). These phages adhere to pili using CoaA protein, specific for a phage species, and upon penetration, ssDNA is converted to circular dsDNA designated as a replicative form (RF), which is used as a template for rolling-circle replication [8]. After production of virion proteins, these phages extrude from cells, forming virions continuously, without causing host death. Besides CoaA protein, key proteins important for phage taxonomy are major coat protein CoaB and Zot protein that forms extrusion pores, and their amino-acid sequences are specific for a genus. The filamentous bacteriophages of *P. aeruginosa* belong to the family *Inoviridae*, and some of them are non-integrative, which replicate exclusively extrachromosomally, while others are integrative, being able to persist as prophages in a host genome [9,10]. The phages Pf1 and Pf3 are two previously described non-integrative filamentous phages of *P. aeruginosa*, that currently are only *P. aeruginosa* filamentous phages that fulfill criteria to be recognized as species by ICTV [8]. Other *P. aeruginosa* filamentous phages are known to be integrated into the genomes of their bacterial hosts: Pf4 and Pf5 persist in the form of prophages in strains PAO1 and UCBPP-PA14 (PA14), respectively [11,12,13]. These two prophages actively produce new virions within their hosts. In addition, PfLES58 prophage from strain LESB58 and Pf7 prophage from strain PA7 are also *P. aeruginosa* integrative filamentous phages, as well as many others in *P. aeruginosa* genomes [14], but they have been poorly examined. 

Alongside Pseudomonas phage Pf1, the most widely studied is Pf4 phage. This phage influences PAO1 biofilm, particularly its formation and dispersal [11,13,15], contributes to the appearance of small colony variants (SCVs) [11], microcolony formation [11], PAO1 *in vivo* virulence [13], promotes PAO1 tolerance to tobramycin, but not to ciprofloxacin [16], enhances resistance to 0.01% SDS [13], decreases swimming [17], and twitching motility [18] etc. However, the data on Pf4 influence on other *P. aeruginosa* strains still remains undetermined, although Pf4 specific genetic elements can be detected in more than 20% of all *P. aeruginosa* strains [14].

The aim of the study was to answer several clinically related questions—whether Pf4 can be induced during antibiotic or phage therapy, whether the released virions are capable to infect other *P. aeruginosa* strains, and how this phage influences virulence factors upon infection of new hosts. Additionally, based on the results, we proposed a new potential approach in phage therapy.

## 2. Materials and Methods

### 2.1. Pseudomonas aeruginosa Strains and Phages

Three strains of *P. aeruginosa* were used in the study: PAO1, LESB58, and UCBPP-PA14 (PA14), all naturally infected with filamentous (pro)phages: Pf4, PfLES58, and Pf5, respectively. The strain PAO1 was used to propagate phage Pf4 and check its induction, while other strains were used as hosts for Pf4 infection. Obligatory lytic phage JG024 was used to induce Pf4. JG024 phage was stored in SM buffer with 10% glycerol at −80 °C. Mueller Hinton (MH) broth (Torlak, Belgrade, Serbia) was used to store the strains, with 10% glycerol at −80 °C. The strains were propagated in MH broth at 37 °C for 24 h or on MH agar, to obtain colonies. Further subcultivation was performed using MH broth, MH agar, Luria Bertani (LB) agar with various percentage of agar, King’s A or King’s B medium, depending on experiment.

### 2.2. Antimicrobial Agents

In this study, discs of ciprofloxacin (CIP, 5 µg), gentamicin (GEN, 10 µg), tetracycline (TET, 30 µg), and streptomycin (STR, 300 µg) (Bioanalyse, Ankara, Turkey) were used. Additionally, the solutions of CIP, GEN, TET, STR, ceftazidime (CAZ), chloramphenicol (CHL), polymyxin B (PMB), and mitomycin C (MMC) (Sigma Aldrich, Saint Louis, MO, USA) were used for MIC determination.

### 2.3. Phage Pf4 Propagation

The strain PAO1 was inoculated in 600 mL LB broth and incubated at 37 °C for 24 h with shaking at 250 rpm. After incubation, the culture was centrifuged at 10,000× *g* at 4 °C for 10 min to remove the bacteria [19]. The phages were precipitated with 4% polyethylene glycol 6000 (PEG6000) (Sigma) and 0.5 M NaCl, and ultracentrifuged in CsCl equilibrium (0.375 CsCl g mL^−1^) using Beckman Ti50 fixed angle rotor (133,000× *g*, 4 °C, 42 h). After ultracentrifugation, the viral band was aspirated and dialyzed repeatedly in SM buffer (2 L of buffer per 1 mL of phage suspension).

### 2.4. Pf4 Induction from Original Host PAO1 

To determine the effect of subinhibitory concentrations of antimicrobials on Pf4 gene expression in PAO1 strain, 1/4 of obtained MIC values were used for all agents (CIP 0.0625 μg mL^−1^, GEN 0.5 μg mL^−1^, CAZ 16 μg mL^−1^, and MMC 0.5 μg mL^−1^). Similarly, PAO1 was treated with 0.25 MOI of JG024 phage (MIC equivalent 1 MOI).

In 50 mL of freshly prepared MH broth, 100 μL of the prepared bacterial suspension was added (0.5 McFarland density; ~10^8^ CFU mL^−1^). The inoculated medium was incubated at 37 °C, 200 rpm, and the optical density of the contents was monitored continuously. After reaching the desired optical density of 0.5 McFarland density, one antimicrobial agent was added to each Erlenmeyer flask, and final volume in each flask was the same. The same volume of sterile distilled water was added in flasks used as negative controls. After 1 h, 6 h, and 24 h of incubation from treatment, samples were taken for bacterial RNA isolation. 

In 50 mL of freshly prepared MH broth, 100 μL of the prepared bacterial suspension was added (0.5 McFarland density; ~10^8^ CFU mL^−1^). The inoculated medium was incubated at 37 °C, 200 rpm, and the optical density of the contents was monitored continuously. After reaching the desired optical density of 0.5 McFarland density, the inoculated medium was treated with JG024. The same volume of SM buffer was added in a flask used as negative control. After 6 h, 12 h, and 24 h of incubation from treatment, samples were taken for bacterial RNA isolation. 

The obtained treatment and control samples were immediately centrifuged at 12,000× *g* for 2 min. RNA was isolated using the GeneJET RNA Purification Kit (Thermo Scientific, Waltham, MA, USA). Concentrations of isolated RNA from all samples were uniformed to avoid variation in growth intensity and treated with DNAse I (1 U) for 30 min at 37 °C, followed by enzyme heat inactivation. 

The isolated RNA was used for reverse transcription using the High Capacity cDNA Reverse Transcription Kit (Applied Biosystems, Bedford, MA, USA) with the following steps: 10 min at 25 °C, then 120 min at 37 °C, and finally 5 min at 85 °C. The reaction mixture was then cooled to 4 °C.

The newly designed primers Pf4Zot and Pf4CoaB (Table S1) were used for the RT-qPCR in triplicate and in three independent repetitions, with the following cycling conditions: an initial cycle of 2 min at 50 °C, the next step at 95 °C for 10 min, then a step at 60 °C for 1 min, followed by a step at 55 °C for 5 s, and the final step lasted 5 min at 95 °C. The Ct values of each sample represent the average + S.E. of the results of replicates. No template controls served as negative controls, and Pf4 DNA served as a positive control. To normalize data, two primer pairs targeting two *P. aeruginosa* housekeeping genes for *rpoD* and *proC* were used (Table S1). Transcriptional changes were calculated using the 2^−ΔΔCT^ method and changes in relative gene expression ≥1.5 or ≤0.67 were considered significant [20].

### 2.5. Pf4 Phage Infection of Other P. aeruginosa Strains

In order to achieve Pf4 infection of LESB58 and PA14, bacteria and phage were mixed at MOI = 10 and incubated overnight at 37 °C. After incubation, the bacteria were centrifuged at 6000× *g* for 2 min. The bacterial pellet was resuspended in PBS buffer and centrifuged again. This step was repeated one more time, to remove unadhered Pf4 virions from the supernatant. Over bacterial pellet, 1 mL of MH broth was added and the bacteria were incubated overnight at 37 °C. Re-washing the bacterial cells and adding freshly prepared medium was repeated twice, to completely remove Pf4 that did not infect bacterial cells, but potentially remained in the medium. The bacteria were then inoculated on MH agar at 37 °C. The subcultivation was repeated three more times in order to obtain stable lysogens.

Upon infection, the presence of Pf4 (pro)phages in different *P. aeruginosa* strains was determined by PCR. Several colonies from each presumed lysogenic strain were used as samples, in such a way that one half of the colony remained on agar in case of successful confirmation of superinfection. Two pairs of primers were used to determine the presence of replicative form (RF) and gene for Pf4 integrase (*intF4*) (Table S1). Thermal cycling conditions for both primer pairs were as follows: an initial cycle of 94 °C for 5 min followed by 35 cycles of 94 °C for 30 s, annealing at 55 °C for 20 s, and extension at 72 °C for 60 s, with a final 7 min extension at 72 °C. PCR products were analyzed by 1.5% agarose gel with ethidium bromide. PAO1 DNA was used as a positive control and sterile distilled water was used as a negative control. 

To further verify the presence of Pf4 (pro)phage in the infected *P. aeruginosa* strains, the obtained PCR products for the RF were cut using enzyme HpaII (FastDigest, Thermo Fisher Scientific, Vilnius, Lithuania). For digestion, 10 μL of PCR product (~0.2 μg), 1 μL of HpaII enzyme, 10 μL of FastDigest Green buffer, and 17 μL of dH_2_O were mixed. The FastDigest mixture was then incubated for 5 min at 37 °C. At the end of the incubation, the restricted PCR products were separated using 1% agarose gel with ethidium bromide. According to in silico analyses, HpaII cut the product of Pf4 RF, giving fragments of 654 and 211 bp.

All gels were documented using BioDocAnalyze Transiluminator (Biometra, Gottingen, Germany).

For confirmation of prophage formation, we postulated that Pf4 was integrated into PA14 and LESB58 using the same *att* site in bacterial genome. We in silico included the phage DNA into bacterial genome and designed primer pairs that comprise both bacterial and viral DNA (Table S1). For purification of PCR products, ExoSAP Master Mix was prepared (100 μL of Exonuclease I (20 U/μL), 200 μL of FastAP (1 U/ μL), 60 μL of Exonuclease I Reaction Buffer 10×, and 240 μL of PCR water). In 5 μL of PCR reaction, 1 μL of ExoSAP Master Mix was added and the samples were incubated for 15 min at 37 °C. Enzyme inactivation was performed at 85 °C for 15 min. For the sequencing purposes, 5 μL of purified PCR product was mixed with 5 μL of forward or reverse primer at a concentration of 5 μM. Sequencing of PCR products was conducted using capillary electrophoresis on an ABI 3730 × l Genetic Analyzer (Applied Biosystems). The alignment was conducted by using DNADynamo program with final manual re-checking. The length of analyzed sequences was 943 bp for both lysogenic strains, LESB58 + Pf4 and PA14 + Pf4.

### 2.6. Production of Pf4 and Indigenous Phages in Superinfected Strains

To confirm the production of Pf4 phage in new hosts of *P. aeruginosa*, lysogenic and non-lysogenic strains were inoculated in 20 mL of MH broth with a final abundance of ~10^6^ CFU mL^−1^. All strains were incubated for 12 h, at 37 °C, at 200 rpm. After 6 h of incubation, 1 mL of bacterial suspensions were used to isolate RNA using GeneJET RNA Purification Kit (Thermo Scientific, USA). The resulting RNA was equalized and purified with 1 μL DNAse I (1 U) for 30 min at 37 °C followed by DNAse inactivation with 1 μL of 50 mM EDTA at 65 °C for 10 min. The purified RNA was translated into cDNA using High Capacity cDNA Reverse Transcription Kit (Applied Biosystems, Bedford, MA, USA) with the following steps: 10 min at 25 °C, then 120 min at 37 °C, and finally 5 min at 85 °C. The reaction mixture was then cooled to 4 °C. The cDNA of all samples was double diluted and then RT-qPCR was used to check the expression of Pf4 phages in lysogenic hosts, as well as the expression of Pf phages that naturally infect given hosts. The primers Pf4CoaA, PfLES58CoaA, and Pf5CoaA (Table S1), specific enough to discriminate all three phages, were designed using PrimerBlast (NCBI) and Oligoanalyzer. To normalize data, two primer pairs targeting two *P. aeruginosa* housekeeping genes were used (*rpoD* and *proC*).

Furthermore, after 12 h of incubation, 10 mL of the bacterial suspensions were purified by centrifugation (6000× *g*, 10 min) followed by filtration through 0.22 μm pores. PEG6000 and NaCl were added to the purified suspensions with final concentrations of 4% and 0.5 M, respectively, and left to incubate overnight at 4 °C. Prior to isolating DNA from the precipitated phages, residual DNA that is not from intact virions was removed by DNase I [21]. Five units of DNase I (≥2500 units mL^−1^) were added to 200 μL of each sample, and then incubated at 37 °C for 10 min. To isolate DNA from phage particles without the need for additional purification steps, DNase I pre-treated phage samples were heat-denatured at 100 °C for 15 min. The viral DNA was then 100-fold diluted and then used for qPCR to compare the production of Pf4 with other Pf phages in newly infected strains. The qPCR primer pairs for Pf4, PfLES58, and Pf5 RF were designed (Table S1).

The qPCR was performed in triplicates and in three independent repetitions, with the following cycling conditions: an initial cycle of 2 min at 50 °C, the next step at 95 °C for 10 min, then a step at 60 °C for 1 min, followed by a step at 55 °C for 5 s, and the final step lasted 5 min at 95 °C. The Ct values of each sample represent the average + S.E. of the results of replicates. No template controls served as negative controls.

### 2.7. Pf4 Influence on Phenotype of Alternative Hosts 

#### 2.7.1. Growth Kinetics 

Prepared bacterial suspensions of 0.5 McFarland density (~10^8^ CFU mL^−1^) of overnight cultures were diluted in PBS buffer at a ratio of 1:100 (*v*/*v*) and then further diluted in a liquid double concentrated MH broth in a ratio of 1:1 (*v*/*v*). Then, 200 μL of inoculated medium was added to the wells of a microtiter plate in triplicates. The microtiter plates were incubated for 24 h at 37 °C in a spectrophotometer with continuous shaking (Thermo Scientific™ Multiskan™ GO) and the absorbance was measured at 600 nm every 30 min. The obtained average values for lysogenic strains were compared with non-lysogenic, original strains. The experiment was conducted in triplicates and results were averaged.

#### 2.7.2. Autoaggregation 

The autoaggregation test was performed by the method of Basson et al., (2007) [22]. The overnight bacterial cultures were aliquoted 1 mL each into sterile tubes and centrifuged for 5 min at 6000× *g*. The resulting supernatant was removed and the cells were resuspended in 1 mL of PBS. These steps were repeated twice. The washed cells were used for the preparation of two series of bacterial suspension. The first series was left untreated containing 800 µL of PBS and 200 µL of resuspended washed bacterial cells, and was prepared prior to measurements. The second series also contained 800 µL of PBS and 200 µL of resuspended washed bacterial cells but it was incubated for 1 h at 37 °C. At the end of incubation, the second series was centrifuged for 2 min at 650× *g*. The resulting supernatant was aliquoted into a microtiter plate in three replicates of 200 µL each. The optical density of aliquoted series was read on a spectrophotometer (Thermo Scientific™ Multiskan™ GO) at 650 nm and the degree of autoaggregation was further determined. The percentage of autoaggregation (%) of the test isolates was determined using the following equation: [(OD0 − OD60)/OD0] × 100, in which OD0 refers to the initial optical density of strains (first series), while OD60 means the optical density obtained after 60 min of treatment (second series). Depending on the degree of autoaggregation, bacterial strains were characterized as highly aggregative (>50%), moderately aggregative (20–50%), and non-aggregative (<20%) [23]. The obtained average values for lysogenic strains were compared with non-lysogenic, original strains. The experiment was performed in at least three independent triplicates.

#### 2.7.3. Cell Surface Hydrophobicity 

Bacterial Adherence to Hydrocarbons (BATH) was used to determine the hydrophobicity of the lysogenic strains [24]. The overnight bacterial cultures (1 mL) were aliquoted into sterile tubes and centrifuged for 2 min at 6000× *g*. The resulting supernatant was removed, and the cells were resuspended in 1 mL of PUM buffer (K_2_HPO_4_ + 4H_2_O 22.2 g, KH_2_PO_4_ + 4H_2_O 7.26 g, urea 1.8 g, MgSO_4_ + 7H_2_O 0.2 g, dH_2_O 1 L). These steps were repeated twice. The washed cells were used to prepare two series of bacterial suspensions. The first series was untreated, containing 900 µL of PUM buffer and 200 µL of resuspended washed bacterial cells. The second series contained 2.7 mL of PUM buffer and 600 µL of resuspended washed bacterial cells (bacterial count was 2 × 10^8^ CFU mL^−1^). The second series was amended with 400 μL of hydrocarbon n-hexane. The tubes were vortexed for 2 min and then left for 15 min at room temperature to separate the phases. The aqueous phase was transferred to new tubes and left in the refrigerator overnight for the hydrocarbons to evaporate. The samples were aliquoted into a microtiter plate in three replicates of 200 µL each. The optical density was read on a spectrophotometer (Thermo Scientific™ Multiskan™ GO) at 560 nm and the degree of hydrophobicity was determined. The percentage of hydrophobicity (%) was determined using the following equation: [(A0 − A1)/A0] × 100, in which (A1) are optical densities of the treated suspensions compared to the suspensions used as a control (A0). Depending on the degree of adhesion to the hydrocarbon, the strains were characterized as extremely hydrophobic (>50%), moderately hydrophobic (20–50%), and hydrophilic (<20%) [24]. The obtained average values for lysogenic strains were compared with non-lysogenic, original strains. The experiment was performed in at least three independent triplicates.

#### 2.7.4. Motility Tests

Twitching, swarming, and swimming motility were assessed by stabbing *P. aeruginosa* strains into 1.5%, 0.7%, and 0.3% agar plates, respectively. After 24 h of incubation at 37 °C, 1.5% and 0.7% LB agar medium, for the determination of twitching and swarming motility of lysogenic and non-lysogenic strains, was removed. Then, to the inside of each Petri dish, 0.4% crystal violet solution was added. In this way, a better visualization of the movement of lysogenic and non-lysogenic strains was achieved. Swimming motility was determined on 0.3% agar plate surface. The experiment was performed in at least three independent triplicates.

#### 2.7.5. Biofilm Formation on Polystyrene Surface

The biofilm formation by *P. aeruginosa* was determined by the method of Knezevic et al., (2008) [25]. The overnight bacterial cultures were aliquoted into sterile cuvettes and centrifuged for 2 min at 6000× *g*. The resulting supernatant was removed and the cells were resuspended in 1 mL of PBS buffer. These steps were repeated twice. The bacterial suspensions of 0.5 McFarland density (~10^8^ CFU mL^−1^) were made. Suspensions were diluted in PBS buffer at a ratio of 1:100 (*v*/*v*) and then further diluted in MH broth in a ratio of 1:1 (*v*/*v*). Then 300 μL of inoculated medium was added to the wells of a microtiter plate. The microtiter plates were incubated for 24 h at 37 °C. After incubation, the medium was carefully removed using a multichannel pipette. The washing of microtiter plate wells was performed twice by 300 µL of PBS. The fixation of the formed biofilm was achieved by adding 300 µL of absolute methanol for 15 min. The methanol was removed and the plate was allowed to dry for 15 min at 44 °C. After that, biofilm was stained by 0.4% crystal violet for 15 min. Afterwards, the microtiter plate was submerged in tap water to remove excess crystal violet. After drying the plate, the dye was dissolved by adding 300 µL of 33% acetic acid. The microtiter plate was left at room temperature for 20 min. The optical density for each well was measured at 595 nm on a spectrophotometer (Thermo Scientific™ Multiskan™ GO). Assessment of cell adhesion was determined using ODc value, which represents the mean of the optical density of the negative control (OD) for the microtiter plate tested, which is increased by three standard deviations obtained for the negative controls. *P. aeruginosa* strains were classified according to the following criteria: OD ≤ ODc = unadherent; ODc < OD ≤ (2 × ODc) = poorly adherent; (2 × ODc) < OD ≤ (4 × ODc) = moderately adherent (4 × ODc) < OD = highly adherent [26]. The obtained average values for lysogenic strains were compared with non-lysogenic, original strains. This test was conducted in triplicates in three independent repetitions.

#### 2.7.6. Pyocyanin Production 

Quantification of produced pyocyanin was determined by using a modified method by Essar et al., (1990) [27]. Bacterial suspensions with an optical density of 0.5 McFarland (~10^8^ CFU mL^−1^) were made from overnight cultures and then further diluted 1:100 (*v*/*v*). To 10 mL of King’s A broth, 100 µL of each strain was added and incubated at 37 °C for 24 h at 200 rpm. After incubation, samples were centrifuged at 6000× *g* for 10 min, and resulting supernatants were collected. To 7.5 mL of supernatant, 4.5 mL of chloroform was added, vortexed 2 × 10 s, and the samples were centrifuged at 4000× *g* for 10 min. Only 3 mL of the resulting blue layer at the bottom was transferred to a new tube. To each tube, 1.5 mL of 0.2 M HCl was added and then vortexed 2 × 10 s. At this step, the blue color turned into pink (top layer). Samples were centrifuged again at 4000× *g*, 10 min, and 1 mL from the pink layer was transferred to a new tube. Spectrophotometric measurements were performed in microplates at 520 nm and pyocyanin concentration (μL mL^−1^) was calculated by multiplying the obtained values and constant 17.072. As a blank, 0.2 M HCl was used. The experiment was performed in triplicates and at least on three independent occasions.

#### 2.7.7. Pyoverdine Production 

Quantification of pyoverdine was determined using a modified method by Déziel et al., (1991) [28]. Bacterial suspensions with an optical density of 0.5 McFarland (~10^8^ CFU mL^−1^) were made from overnight cultures and then further diluted 1:100. To 2 mL of King’s B Medium, 100 µL of each strain was added and incubated at 37 °C for 24 h at 200 rpm. At the end of the incubation, 1 mL was used to determine the total number of bacteria. The residual overnight cultures were centrifuged and the resulting supernatants were used to quantify the relative concentration of pyoverdine by measuring the fluorescence (Fluoroscan Ascent FL, Thermo Labsystems, Waltham, MA, USA) at 460 nm after excitation with wavelength 340 nm. The experiment was performed in triplicates and at least on three independent occasions.

#### 2.7.8. SCV Production

Non-lysogenic and Pf4 lysogenic strains were inoculated into 5 mL of freshly prepared Luria–Bertani broth and incubated at 37 °C for 5 days without agitation. At the end of the incubation, the bacterial suspensions were centrifuged (6000× *g*, 5 min) and the resulting pellet was resuspended in 2 mL of PBS. This washing step was repeated once more, and then serial dilutions were made. The total numbers of wild-type and SCVs’ colonies for each strain were determined by the spread plate method on Luria–Bertani agar with 0.04% Congo Red [29]. Colony counting was performed after 2 days of incubation at 37 °C. The experiment was conducted in triplicates and results were averaged.

### 2.8. Antibiotic Susceptibility

For preliminary estimation of antibiotic sensitivity of uninfected and Pf4-infected *P. aeruginosa* strains, a disk diffusion method was applied using discs of CIP, GEN, TET, and STR [30].

The antibiotic susceptibility of lysogenic strains was further determined by determination of minimal inhibitory concentration (MIC). Prepared bacterial suspensions of 0.5 McFarland density (~10^8^ CFU mL^−1^) of overnight cultures were diluted in MH broth (1:100 *v*/*v*). Then 100 μL of inoculated medium was added to the wells of a microtiter plate. The same volume of antibiotics was added to the wells, with the final concentration of each antibiotic in the microtiter plates ranging from 0.0625 to 128 μg mL^−1^. The following antibiotics were used: CIP, GEN, STR, TET, CAZ, CHL, and PMB. Total bacterial counts in each well of the microtiter plate were ~1 × 10^6^ CFU mL^−1^. The microtiter plates were incubated for 18 h at 37 °C, after which 10 μL of 0.1% solution of 2,3,5-triphenyltetrazolium chloride (TTC) was added to each well of the plate, which was reduced to red formazan by the dehydrogenases of viable bacterial cells. The microtiter plates were further incubated for 2 h at 37 °C, after which the MIC value for each antibiotic was read. The lowest concentration of antibiotics needed to prevent coloring of TTC to red formazan was considered as an MIC value. The reference strain *Escherichia coli* ATCC 25922 was used as a control. The experiment was performed in at least three independent triplicates. The obtained results are presented as geometric mean. The reduction of MIC after phage infection by one value was not taken as statistically significant. Lysogenic and non-lysogenic strains have been characterized as sensitive, intermediate sensitive or resistant to antibiotics, tested according to the recommended criteria for *P. aeruginosa* or, if not available, for other non-enterobacteriace (for TET, STR, and CHL) [31].

### 2.9. Statistics

To compare virulence factors of non-lysogenic and lysogenic strains, Statistica 7.1 software was used. Normality of data distribution was determined by Kolmogorov–Smirnov Test of Normality. Difference of normally distributed data were tested by parametric paired *t*-test, while data without normal distribution were analyzed by Wilcoxon signed rank test. Confidence interval was CI95, and *p* values <0.5 were considered statistically significant.

## 3. Results

### 3.1. Can Pf4 Be Induced from Original PAO1 Strain by Subinhibitory Concentration of Antibiotics or Infection by an Obligatory Lytic Phage at Low MOI?

**Figure 1:**
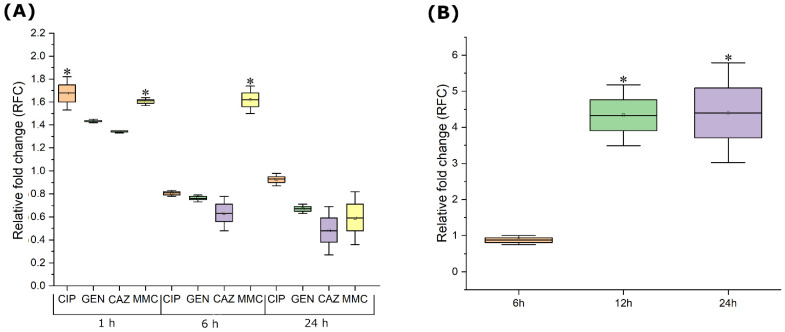
Change of Pf4 *coaB* and *zot* gene expression in PAO1 strain after (**A**) treatment with ¼ MIC of CIP (light orange), GEN (green), CAZ (purple), and MMC (light yellow) and (**B**) infection with phage JG024 at MOI 0.25 (equivalent to ¼ MIC). Results are average + S.E., n = 6; calculated using 2^−ΔΔCT^ method, and cut-off for relative change in expression was ≥1.5 or ≤0.67; * ≥1.5.

Induction of Pf4 from original host PAO1 was examined upon bacterial treatment by 1/4 MIC of antimicrobials, by qRT-PCR examination of *coaB* and *zoT* gene expression. The treatment with 1/4 MIC of ciprofloxacin showed a change in the expression of key Pf4 genes after 1 h (approx. RFC 1.68), but not after 6 and 24 h (Figure 1A). In contrast to ciprofloxacin, RFC for gentamicin and ceftazidime treatment remained below 1.5 during 24 h of incubation, indicating no significant change in Pf4 gene expression. Pf4 induction by mitomycin C was significantly higher after 1 and 6 h upon exposure (RFC 1.61 and 1.62, respectively). However, after 24 h, for all four antimicrobial agents, RFC was close to 1 or even slightly lower. 

Induction of Pf4 from the original host PAO1 was also examined upon infection by obligatory lytic phage JG024, by examining expression of the same genes as for antibiotics (Figure 1B). After 6 h of treatment with 0.25 MOI of JG024, no statistically significant change in the expression of key Pf4 prophage genes was observed. However, the RFC was significantly higher after 12 h and 24 h of incubation, being at least 4 times higher.

### 3.2. Can Pf4 Infect Model Strains PA14 and LESB58?

**Figure 2:**
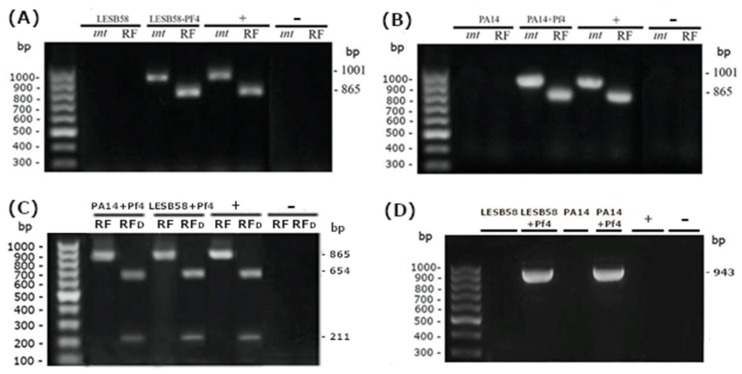
Confirmation of Pf4 infection of LESB58 (**A**) and PA14 (**B**) strains. Primer pairs targeting Pf4 integrase gene (*intF4*) and replicative form (RF) were used, and expected products were obtained for Pf4 phage (bp). The obtained product for Pf4 RF was digested with HpaII enzyme (**C**) giving expected products of 654 and 211 bp. Confirmation of Pf4 bacteriophage integration in the genome of LESB58 and PA14 (**D**). The strain PAO1 was used as a positive control (+) and sterile distilled water as a negative (−).

Both *P. aeruginosa* strains LESB58 and PA14 harboring indigenous filamentous phages (PfLES58 and Pf5) can be successfully infected with Pf4 phages. This was confirmed by detection of Pf4 integrase (*intF4*) and replicative form (Pf4RFc) (Table S1) upon infection, while these elements could not be detected in the wild-types of these two strains (Figure 2A,B). The specificity of the RF PCR product was confirmed by HpaII digestion and fragments of expected sizes were obtained (Figure 2C). Furthermore, the assumed Pf4 *att* site was confirmed by sequencing of corresponding PCR products (Access No. ON398428 and ON398429; Table S2), obtained using primer pairs (Pf4/LESB58 and Pf4/PA14) (Table S1) that cover both bacterial DNA (a part of the gene for probable transferase), *att* (tRNA-Gly), and phage genomes (a hypothetical gene). The results indicate that DNA of Pf4 is integrated in the bacterial genomes as prophages, in both cases near tRNA for glycine (Figure 2D).

### 3.3. How Does Pf4 Change Production of the Indigenous Filamentous Prophages of Lysogenic Strains?

**Figure 3:**
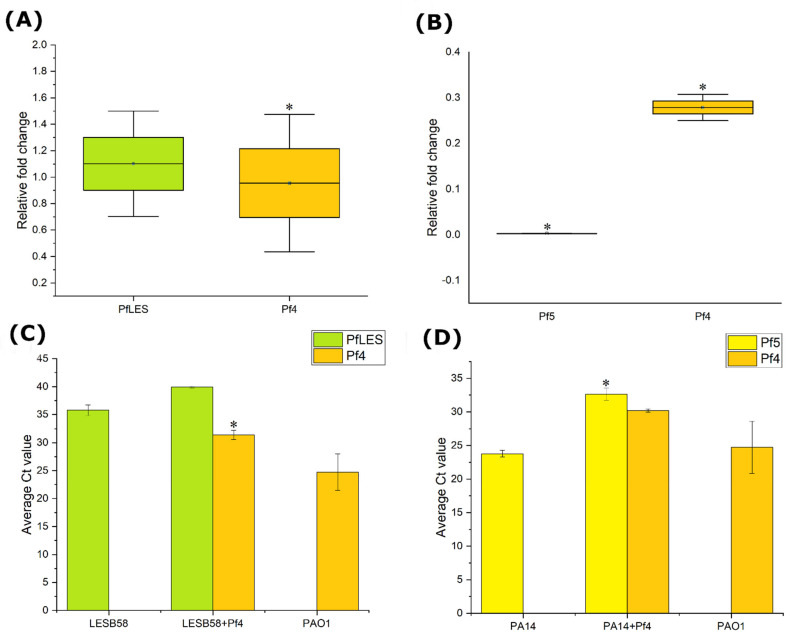
qRT-PCR analysis of Pf4 and indigenous phage: (**A**,**B**) *coaA* expression at RNA level in LESB58 and PA14 strains after 6 h of incubation, respectively. Results are average + S.E; n = 6; calculated using 2^−ΔΔCT^ method, and cut-off for relative change in expression is ≥1.5 or ≤0.67. * ≥1.5 or ≤0.67 (**C**,**D**) qPCR analysis of Pf4 and indigenous phage production at viral DNA level in LESB58 and PA14 strains using RF of bacteriophages after 24 h of incubation, respectively. Results are average Ct values + S.E., n = 6, calculated using Student’s *t*-test; * *p* ≤ 0.05.

Detecting *coaA* gene expression and RF presence in bacterial DNA by qRT-PCR, it was confirmed that both indigenous prophages, Pf5 from PA14 or PfLES58 from LESB58, are still produced upon infection with Pf4. Moreover, Pf4 is also produced from the infected corresponding strains along with Pf5 or PfLES58. Following infection of the LESB58 strain with Pf4 phage, there was no significant change in PfLES58 phage expression (Figure 3A). On the other hand, in PA14 + Pf4, a significant decrease in Pf5 gene expression was observed in the presence of Pf4 phage (Figure 3B). The RFC for this strain averaged 0.003 (more than 300 times). The PAO1 strain was used as a control. The expression of Pf4 phage in the PA14 strain was statistically significantly reduced (Figure 3A) compared to PAO1, while in the LESB58 strain it remained unchanged (Figure 3B).

qPCR analysis of isolated viral DNA confirmed the presence of Pf4 and PfLES58 phages in the lysogenic LESB58 strain, and Pf4 and Pf5 phages in the lysogenic PA14 strain, respectively. PfLES58 phage production after Pf4 phage infection remained stable and without significant statistical changes (Figure 3C). In the non-lysogenic LESB58 strain, the Ct value for RF PfLES58 phage was 28.96 ± 0.50, while in the Pf4 lysogenic strain it was 32.94 ± 0.37. Significantly lower production of Pf5 phages was observed in lysogenic strain in the presence of Pf4 phages (Figure 3D). In the non-lysogenic PA14 strain, the Ct value for RF Pf5 phage was 23.79 ± 0.5 compared to the Pf4 lysogenic strain where the Ct value was 32.63 + 0.9. These results coincide with the relative change in PfLES58 and Pf5 phage gene expression at the RNA level in both lysogenic strains. The PAO1 strain was used as a positive control and the Ct value of Pf4 in that strain averaged 24.73. However, in both lysogenic strains, the production of Pf4 phage was lower. In PA14 + Pf4, it was 29.96 ± 3.75 (Figure 3D), and in LESB58 + Pf4, it was 31.39 ± 0.83, which is a statistically significant difference (*p* < 0.05) (Figure 3C). 

### 3.4. Can Pf4 Influence Biological Properties and Virulence Factors of Lysogenic Strains?

#### 3.4.1. Growth Kinetics

**Figure 4:**
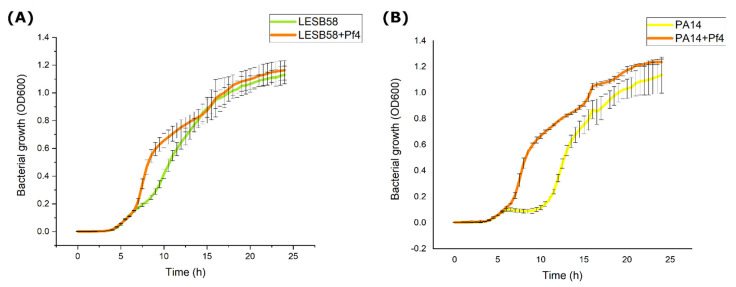
Growth of uninfected and Pf4-infected LESB58 (**A**) and PA14 (**B**) strains, monitored every 30 min during 24 h, and expressed as OD600 (average + S.D.).

The Pf4 slightly influenced growth kinetics of both examined strains, as presented in Figure 4. The phage Pf4 slightly stimulated growth of both strains between 7 and 15 h for LESB58 and 7 and 21 for PA14, but thereafter, both infected and uninfected strains reached the same OD values. 

#### 3.4.2. Autoaggregation 

**Figure 5:**
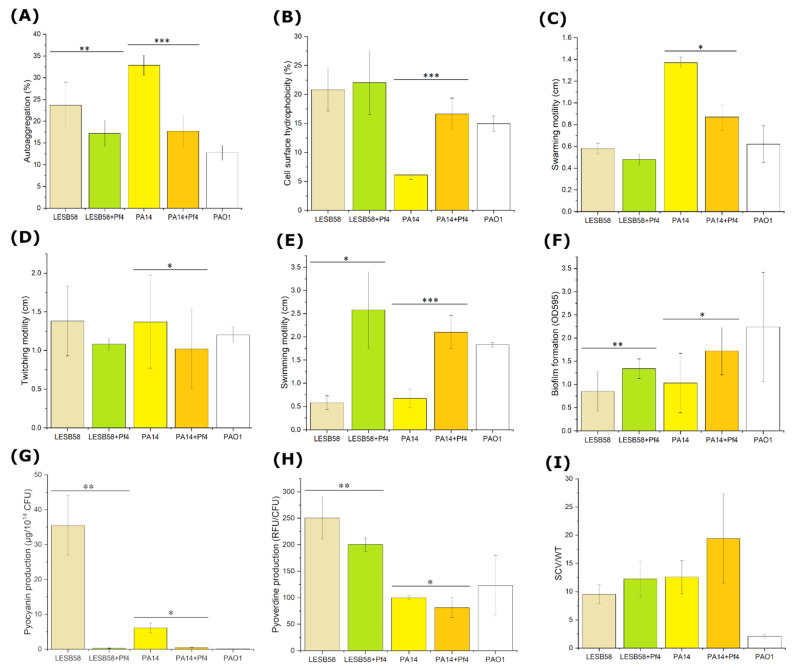
Changes in autoaggregation (**A**), cell surface hydrophobicity (**B**), swarming motility (**C**), twitching motility (**D**), swimming motility (**E**), biofilm production (**F**), pyocyanin (**G**) pyoverdine production (**H**), and prevalence of SCV (**I**) of Pf4-infected and uninfected LESB58 and PA14 strains. The results are average + S.E. n = 9; *** *p* < 0.001; ** *p* <0.01; * *p* < 0.05; tested by Wilcoxon signed rank test.

Pf4 lysogenic and non-lysogenic strains showed different autoaggregation abilities. Namely, upon Pf4 infection, both LESB58 and PA14 strains changed autoaggregation ability from moderately aggregative to non-aggregative (*p* < 0.001 and 0.0001, respectively) (Figure 5A). 

#### 3.4.3. Cell Surface Hydrophobicity

Pf4 enhanced hydrophobicity of both strains, but a significant increase (*p* < 0.001) was observed only in PA14 (Figure 5B). Still, the strain remained hydrophilic. 

#### 3.4.4. Motility of Lysogenic Strains 

Presence of Pf4 phages in both infected strains contributed to higher swimming motility on semi-solid medium (Figure 5E). In the case of Pf4 lysogeny, a radius of strain motility ranged from 2.1 cm up to 2.58 cm on average. Non-lysogenic strains barely reached 0.7 cm during the same period of incubation. The difference was statistically significant for both strains (*p* < 0.0001 or *p* < 0.05). The phage Pf4 lysogenization reduced the swarming motility in both *P. aeruginosa* strains (Figure 5C), and in PA14 + Pf4 strain, the difference was significant (*p* < 0.01). The infection with Pf4 reduced twitching motility, with statistically significant difference, only for Pf4-infected PA14 (*p* < 0.05) (Figure 5D).

#### 3.4.5. Biofilm Formation on Polystyrene Surface 

The infection of Pf4 phages in different *P. aeruginosa* strains resulted in a larger amount of formed biofilm. In both strains, biofilm production was greater than in non-lysogenic (*p* < 0.05 or *p* < 0.01) (Figure 5F). 

#### 3.4.6. Pyocyanin Production by Lysogenic Strains

The influence of Pf4 filamentous phage on the production of pyocyanin, an important *P. aeruginosa* virulence factor, was inhibitory in both strains, with statistical significance (*p* < 0.01 or *p* < 0.05) (Figure 5G).

#### 3.4.7. Pyoverdine Production by Lysogenic Strains

Similar to pyocyanin production, Pf4 phages affected pyoverdine production of hosts. Pf4 phages achieved the same effect in both lysogenic strains, and pyoverdine production was generally reduced by approx. 20%, with statistical significance (*p* < 0.01 or *p* < 0.05) (Figure 5H).

#### 3.4.8. SCV Production

After 5 days of incubation, an increased appearance of SCV was observed in both *P. aeruginosa* strains after Pf4 infection but with no statistically significant difference (*p* > 0.05) (Figure 5I).

### 3.5. Antibiotic Susceptibility

**Figure 6:**
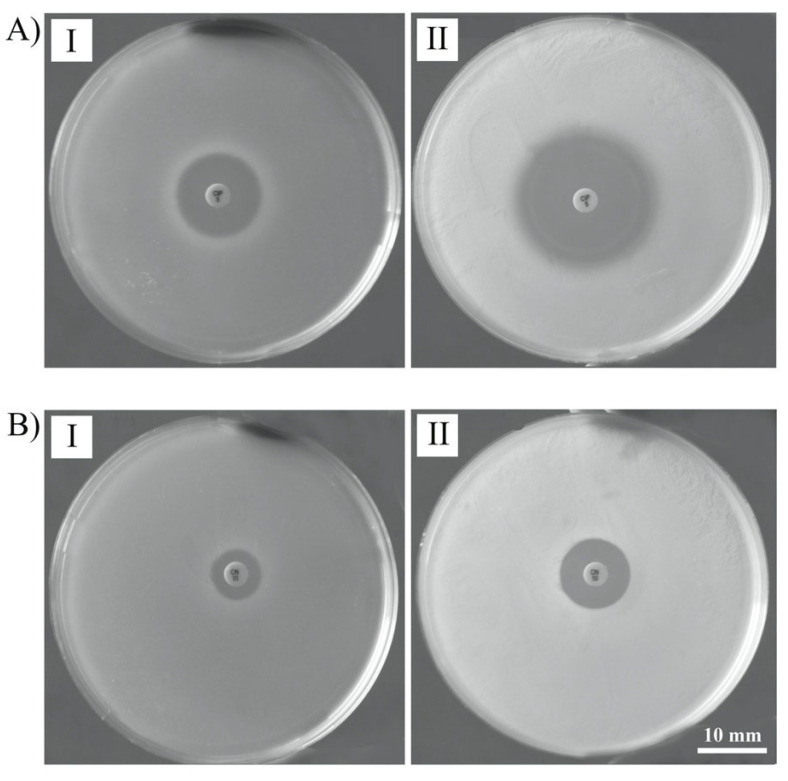
Changes in susceptibility of Pf4 uninfected (**I**) and PF-infected (**II**) strain LESB58 to CIP (**A**) and GEN (**B**).

To examine Pf4 influence on lysogeny antibiotic susceptibility, both disk diffusion and MIC methods were used. Pf4 lysogenic LESB58 showed increased susceptibility to ciprofloxacin using disk diffusion method (approx. an increase in inhibition diameter 6.5 mm), but changes were also obvious for gentamicin (3 mm) (Figure 6(AII) and Figure 6(BII), respectively). Based on these preliminary findings, a wide range of antibiotics from different classes were tested against lysogenic and non-lysogenic strains to determine MIC values. Filamentous phage did not contribute to the increased resistance of their hosts to the used antibiotics (Table 1). Pf4 phages made the LESB58 strain more sensitive to fluoroquinolones, aminoglycosides, and β-lactams. The MIC for the LESB58 non-lysogenic strain for CIP was 0.5 μg mL^−1^, and in the presence of Pf4, it decreased to 0.125 μg mL^−1^. The same was observed in the case of GEN and STR, also showing the increase of susceptibility by two MIC values. The largest difference was observed for CAZ, because the MIC in the LESB58 strain was greater from 64 μg mL^−1^, while MIC for infected strain was only 16 μg mL^−1^. Pf4 also re-sensitized PA14 to fluoroquinolones and the MIC decreased from 0.125 to <0.00625 μg mL^−1^.

## 4. Discussion

One the best known *P. aeruginosa* strains is PAO1, which harbors an integrative filamentous phage Pf4. Its influence on original host phenotypic properties is well established. Here, we examined inducibility of this phage, its possibility to infect other *P. aeruginosa* strains, and Pf4 contribution to their virulence factors.

The phage Pf4 can be produced in greater amounts, approx. more than 50%, after exposure to subinhibitory concentrations of ciprofloxacin and mitomycin C. While ciprofloxacin increased Pf4 phage production only briefly after PAO1 exposure to subinhibitory concentrations, mitomycin C had prolonged activity up to 6 h. Since both antimicrobials triggered the SOS response [32,33], they probably affected Pf4 production by the same mechanism. The reason why this induction is only contemporary may be related to the bacterial adaption to a stressful environment and SOS response termination [32]. Mitomycin C has been previously used to test the induction of Pf4 phages of PAO1 strain, and it was found that the treated strain released superinfective Pf4 forms, determined by plaque assay, after the use of mitomycin C in concentrations higher than 10 μg mL^−1^ [15]. Accordingly, both ours and previous results indicated that Pf4 belongs to the group of mitomycin C inducible phages. On the other hand, gentamicin and ceftriaxone were not able to enhance Pf4 production and they were unable to activate SOS response, so this additionally supports the assumption related to SOS response. Moreover, for the first time, we proved that the infection of PAO1 with obligate lytic phage JG024 at MOI 0.25 can affect the expression of Pf4 prophage key genes up to 24 h after infection. Namely, this MOI could not cause complete lysis of bacteria during 24 h, probably due to the appearance of phage- resistant mutants. Thus, in such a system, phage Pf4 production is enhanced up to four times, probably as a result of altered metabolism of the cells and Pf4 regulatory mechanisms in phage-resistant, i.e., mutated cells. The results indicate that ciprofloxacin, mitomycin C, and obligatory lytic phages can induce filamentous phages during therapy and that they should be applied with precautions. 

Strains of *P. aeruginosa* LESB58 and PA14 are naturally infected with filamentous phages PfLES58 and Pf5, respectively, and can successfully be superinfected with Pf4 phages. The presence of Pf4 RF in these strains, as well as in the isolated viral DNA, indicates that this phage established a chronic productive infection in the given strains. Alongside the persistence of phage Pf4 in the RF, i.e., extrachromosomally, the sequencing results indicate that Pf4 was integrated in the genome of PA14 and LESB58 as a prophage in the proximity of tRNA-Gly, as in PAO1. The successful infection of the strains by Pf4 is not surprising, as it has been previously documented that one bacterial strain can carry several different or even the same prophages from the family *Inoviridae* [14]. Since all *Inoviridae* utilize pili as receptors, it is expected that piliated cells, including PA14 and LESB58, are prone to infection by filamentous phages. The superinfection exclusion was not observed between PfLES58 or Pf5 and phage Pf4. PfLES58 phage expression in the presence of Pf4 phage was not significantly altered, while in the case of Pf5 phage, the change was drastic—Pf4 phage significantly reduced the expression and production of Pf5 virions in the PA14 + Pf4 strain. Pf4 phage expression in the first 6 h of incubation was also statistically significantly reduced. However, viral DNA analyses indicated that Pf4 phages reached a similar number of virions relative to PAO1, while Pf5 phage production remained reduced. In LESB58 + Pf4, the expression of both phages was unchanged, but PfLES58 predominated in virion production, and there was a statistically significant decrease in Pf4 virion in this lysogenic strain. This indicates that Pf4 virions, released increasingly during therapy, can successfully infect new strains of *P. aeruginosa*, establishing very complex interactions with other indigenous filamentous (pro)phages.

The phage Pf4 influence on PAO1 phenotype is well documented in the literature, and here it was confirmed that it also changed virulence factors of alternative hosts PA14 and LESB58.

Filamentous phages usually reduce bacterial growth, as reported for *Xanthomonas citri* [34,35], *Xanthomonas campestris* [36], *Ralstonia solanacearum* [37], *Pseudoalteromonas* [38], *Vibrio alginolyticus* [39], and *E. coli* [40,41]. However, there are known cases where filamentous phages do not affect the growth of their host as in *X. campestris* [42], *R. solanacearum* [43], and *Yersinia pestis* [44]. Here, we showed for the first time, that filamentous phages can slightly increase bacterial growth, but only contemporarily. The potential explanation is related to the fact that Pf4 encoded a toxin-antitoxin system type II, having an antitoxin with conserved sequence Phd_YeFM [45]. This antitoxin binds to their toxin partners, can bind DNA via the N-terminus and repress the expression of operons containing genes encoding the toxin and the antitoxin. This domain complexes with Txe toxins with various domains, and is present in *P. aeruginosa* cells [46], which can partially explain the slight stimulation of growth. After 24 h incubation, the difference in growth cannot be observed, so the Pf4 infection has no long-term influence on PA14 and LESB58 growth. 

The ability of cells to aggregate can play an important role in the initial phases of biofilm formation. Addy et al., (2012) [47] hypothesized that the presence of filamentous phages on the surface of a bacterial cell may contribute to changes in the nature of the cell membrane, and increase cell to cell interactions which would contribute to high local cell densities. However, it seems the opposite happened with PA14 and LESB58 *P. aeruginosa* cells upon infection with Pf4 phage, as autoaggregation was decreased—non-lysogenic strains were moderately aggregative, but after infection with Pf4 phage, they became non-aggregative. 

Upon the phage Pf4 infection, a slight increase in hydrophobicity in both examined lysogenic strains was observed, similar to findings for Ralstonia phage φRSS1 [47]. However, the lysogenic strains remained either hydrophilic or moderately hydrophobic, i.e., without qualitative change in hydrophobicity. The increase in hydrophobicity can be explained by the fact that upon filamentous phage infection, lipids of the outer membrane are altered with change of the relative concentration of phospholipids [48]. 

Several cell surface-associated structures, such as flagella and type IV pili, are essential for adhesion and microcolony formation, respectively [49,50]. Flagella act to overcome repulsive forces between the bacterium and the surface to allow the initial contact [51]. This is the first report on increased swimming motility upon a filamentous phage infection that correlates with increased biofilm production, since bacterial motility plays an important role in the initial stages of biofilm formation [52]. This finding is very interesting, since difference in swimming motility was not previously established for PAO1 strain infected with Pf4 and deprived of the prophage [17]. Similar lack on swimming motility influence was observed for other phages, such as phage SW1 of *Shewanella piezotolerans* [53]. On the other hand, *Xanthomonas* cells infected with XacF1 showed reduced swimming activity [34]. As this is the first report on swimming motility increase upon filamentous phage infection, this phenomenon deserves further elucidation. 

Both swarming and swimming motility are a flagellum-dependent form of movement observed in *P. aeruginosa*. Overlapping sets of regulators have been found to affect swarming motility and biofilm formation in a reciprocal manner [54,55]. For example, strains lacking GacA, responsible for biofilm formation and EPS production, had increased swarming motility [56], while strains with increased EPS production had reduced swarming motility [54]. The presence of Pf4 in *P. aeruginosa* strains led to a decrease in swarming motility in both strains, with significant difference observed for Pf4-infected PA14 strain. A similar example of decreased swarming motility caused by filamentous phages can be found in *Erwinia amylovora* infected with PEar filamentous phages [57], and *S. piezotolerans* infected with SW1 phage [53]. Together, these results confirm that the presence of filamentous phages may reduce swarming motility in different bacterial species, probably by change in the expression of key genes responsible for this type of motility.

Using type IV pili, bacteria exhibit twitching motility, which is movement over solid surfaces [58]. The twitching motility is considered an important bacterial property that leads to grouping of cells into microcolonies [49]. Furthermore, type IV pili are bacterial adhesins significant for bacterial interaction with mammalian cells, but also for autoaggregation. As indicated, filamentous phages use these structures as receptors, when establishing infection of bacteria [59]. The results of the present study showed that in the presence of Pf4, there was a slight decrease in twitching motility in both lysogenic strains, with statistically significant difference observed for PA14 strain. This is not the first report regarding Pf phages and reduction of twitching motility. Secor et al., (2017) [18] demonstrated that PAO1 strain superinfected with Pf4 phage exhibited minimal twitching motility. Similarly, filamentous phages of other bacterial species also reduced twitching motility, such as Xanthomonas phage XacF1 [34], Ralstonia phage RSS51 [43], and Ralstonia phage RSM3 [60]. The strains used in this study, being naturally infected with other types of filamentous phages, produced Pf4 phage in addition, so the numerous phages can reinfect cells adsorbing to pili and impair their function. Another possibility is that Pf4, directly or indirectly, shuts down genes for pili production, to prevent superinfection by other similar phages, which should be further examined. The lysogenic strains with Pf phages are thought to be less invasive due to impaired twitching motility and to have a phenotype similar to twitching-deficient non-invasive *P. aeruginosa* phenotypes [61]. Finally, this finding is in correlation with the detected decrease in autoaggregation. 

Bacteria often switch from a free-living lifestyle to a surface-adapted multicellular lifestyle known as a biofilm. It has been estimated that 60% of all *P. aeruginosa* infections are biofilm related [6,62], and there is substantial evidence that biofilm plays an important role in persistent infections, e.g., lungs in CF patients. Our results show that *P. aeruginosa* Pf4 lysogenic strains formed more quantity of biofilm compared to their non-lysogenic counterparts. For PAO1 strain, it is well documented that Pf4 phage plays a crucial role in biofilm formation and persistence [11,13,16]. Similar to type IV pili, which facilitate bridging and permanent attachment of cells to the surface, the presence of a larger number of filamentous phages on the surface of the bacterial cell could have the same impact on biofilm formation [11,63], contributing to microcolony expansion [13]. However, this cannot simply explain our results, as autoaggregation was decreased in Pf4-infected strains, and the same pertains to twitching motility, both considered important to biofilm formation. The enhanced cell surface hydrophobicity and swimming motility also contributed to cells’ better interaction with surface, and the established biofilms were probably further more intensively developed, as numerous phage particles contributed to the biofilm matrix structure. The role of Pf4 in biofilm matrix structure [13,16], as well as in biofilm volume and thickness [64] were previously documented for PAO1 strain. Cell lysis by Pf4 phage may additionally contribute to better biofilm production, and this phenomenon was confirmed in the original host PAO1 [15]. The cell lysis released nutrients into the biofilm matrix [65,66], while released eDNA contributed to matrix structure [67]. Accordingly, the strains infected with Pf4 phage produced more quantities of biofilm than uninfected counterparts, probably not because of enhanced initial adhesion and more efficient microcolony formation, but rather due to a more complex biofilm matrix architecture which occurred in later phases of biofilm development.

Pyocyanin is a human cytotoxin, which is considered to be an extremely important secreted virulence factor of *P. aeruginosa* [68]. Infection by Pf4 phages severely inhibited pyocyanin production of lysogenic strains. Ismail et al., (2021) [69] demonstrated that PAO1 without Pf4 phage produced less pyocyanin compared to wild-type after 24 h of incubation. Although it seemed that Pf4 stimulated pyocyanin production in its original host, it decreased pyocyanin production in PA14 and LESB58 *P. aeruginosa* strains. Pyoverdine is a water soluble siderophore, i.e., iron-chelating molecule, able to trigger activation of virulence factors, exotoxin A and protease PrpL, when it is in the form of ferripyoverdine [70]. A significant decrease in pyoverdine production was observed in both lysogenic strains. Similarly, the PAO1 mutant in the absence of Pf4 prophage was shown to produce significantly more pyoverdine than its wild-type [69]. The results obtained for *P. aeruginosa* exopigment production suggest that the Pf4 decreased toxicity of the examined strains.

Small colony variant (SCV) is a phenotypic variant of *P. aeruginosa* colonies, which are circular opaque dwarf colonies with a diameter about three times smaller than wild-type colonies, frequently obtained from samples of *P. aeruginosa*-infected cystic fibrosis lung [71]. The phage Pf4 is known to cause small colony variant (SCV) at the late stage of PAO1 biofilm development [11,13]. Moreover, increased *xisF4* gene expression in PAO1 can lead to higher Pf4 phage production, and then the number of SCV colonies increased by 55% [72]. However, the percentage obtained in this study for SCV in lysogenic strains was below 10%, with no significant difference. Interestingly, PA14 strain carries Pf5 prophage, and its relatedness to SCV formation had not been proven previously [12]. Based on the previous finding that SCVs lack twitching motility [53], and the results that Pf4 decreases twitching motility, we expected to detect a higher percentage of SCV, which was not the case. It was obvious that Pf4 did not influence SCV production in PA14 and LESB58. 

The infection with Pf4 filamentous phages can contribute to the increased antibiotic susceptibility of bacteria, and the phenomenon seems to be both strain- and antibiotic-dependent. Namely, the increase in antibiotic activity was obvious for ciprofloxacin, gentamicin, ceftazidime, and streptomycin, against Pf4-infected LESB58, and for ciprofloxacin and ceftazidime against Pf4-infected PA14. It is interesting to notice that sensitivity of both LESB58 and PA14 to ceftazidime changed from resistant to intermediately sensitive after Pf4 infection, indicating significant re-sensitization to this antimicrobial agent. Moreover, LESB58 changed sensitivity to streptomycin upon Pf4 infection from intermediately sensitive to sensitive, while in PA14, this change was from resistant to intermediate resistant. Finally, original LESB58 was intermediately sensitive to tetracycline, and became sensitive upon Pf4 infection. 

As Pf4 contributes to phage enhanced growth in a form of biofilm and knowing that bacteria in biofilm are up to 1000 times resistant [73], it can be expected that sensitivity will decrease, but results indicated the opposite. However, the method comprises immediate treatment of planktonic bacteria with antibiotics, so the antibiotic exhibits its activity prior to possibility of biofilm formation. The increased sensitivity upon filamentous phage infection was previously documented for other phage-host systems; e.g., Wang et al., (2013) [74] found that Vibrio phage VEJ increased *Vibrio cholerae* sensitivity to ampicillin. Hagens et al., (2006) [75] indicated that filamentous phages in combination with small doses of antibiotics can inhibit and even kill *P. aeruginosa* strains. Moreover, two different PAO1 strains, one harboring a gentamicin resistance plasmid and second to tetracycline, became more susceptible after the use of filamentous phages in combination with gentamicin and tetracycline. The authors hypothesized that pores for extrusion influence the host cell membrane permeability, so the membrane is less effective as a barrier against antibiotic penetration. Although this explanation seems reasonable, the pores for extrusion contain a multimeric tightly-gated channel through the membrane that does not allow molecule inflow into the cell [76]. The cryo-electron microscopy revealed that the multimeric channel has a central pore 6.0–8.8 nm in diameter, but in the middle of the channel, the pore is tightly closed [77]. The finding that susceptibility is increased for various antibiotics, however, supports the assumption that Pf4 phage changes cell permeability upon infection. Thus, another possible mechanism is that *Pseudomonas* filamentous phages can change the membrane potential of cells, previously documented for Escherichia phage f1 [78]. This change can consequently alter membrane permeability by affecting the function of certain channels in the outer membrane, as a result of phage protein integration into membranes prior to extrusion. Moreover, it can lead to the opening of channels for phage extrusion, allowing antibiotic inflow into cells. If the extrusion pores can be opened prior to phage extrusion, and if they can allow molecule inflow into a cell, should be revealed in future studies. Our findings open up a new possible approach in phage therapy—treatment of multidrug and pan drug resistant strains with filamentous phages, in order to re-sensitize bacteria to certain antibiotics. However, this approach has to be considered with caution in order to avoid unwanted lysogenic conversion and increased virulence of strains. The choice of phage should be focused on those that do not contribute to the formation of biofilm and that significantly increase the susceptibility of strains to antibiotics.

## 5. Conclusions

In summary, the results clearly indicate that Pf4 can be induced by subinhibitory concentrations of certain antimicrobial agents or by infection by obligatory lytic phages, and can successfully infect other strains. In these strains, Pf4 can establish a chronic productive infection with integration into the host genome, and expression of Pf4 genes can exceed expression of indigenous filamentous prophages. Pf4 phages influence many virulence factors of newly infected *P. aeruginosa*, mainly by decreasing virulence, with exceptions of swimming motility and biofilm production. Finally, Pf4 infection can re-sensitize newly infected strains to antibiotics, which potentially has great therapeutic implications. 

## Figures and Tables

**Table 1:** MIC of antibiotics for uninfected and Pf4-infected *P. aeruginosa* strains.

*P. aeruginosa* Strains	MIC (μg mL^−1^) ^1^						
	CIP	GEN	TET	CAZ	STR	CHL	PMB
LESB58	0.5 (S) ^2^	0.5 (S)	8.0 (I)	>64.0 (R)	64.0 (R)	32.0 (R)	1.0 (S)
LESB58 + Pf4	0.125 (S)	0.125 (S)	4.0 (S)	16.0 (I)	16.0 (I)	32.0 (R)	1.0 (S)
PA14	0.125 (S)	0.5 (S)	16.0 (R)	64.0 (R)	16.0 (I)	64.0 (R)	1.0 (S)
PA14 + Pf4	<0.0625 (S)	0.5 (S)	16.0 (R)	16.0 (I)	8.0 (S)	32.0 (R)	0.5 (S)
PAO1 ^3^	0.0625 (S)	1.0 (S)	8.0 (R)	16.0 (I)	16.0 (S)	32.0 (R)	1.0 (S)
*E. coli* ATCC 25922	<0.0625 (S)	1.0 (S)	1.0 (S)	1.0 (S)	8.0 (S)	4.0 (S)	0.25 (S)

^1^ CIP—ciprofloxacin; GEN—gentamicin; TET—tetracycline; CAZ—ceftazidime; STR—streptomycin; CHL—chloramphenicol; PMB—polymyxin B. ^2^ (R)—resistance; (I) intermediate resistance; (S)—sensitive. ^3^ Determined for bacterial count 1 × 10^8^ CFU mL^−1^.

## Data Availability

Both sequences obtained from the LESB58 + Pf4 and PA14 + Pf4 genomes have GenBank accession numbers ON398428 and ON398429, respectively.

## Supplementary Materials

The following supporting information can be downloaded at: https://www.mdpi.com/article/10.3390/v14061261/s1, Table S1: Primers used in the study and Table S2: Sequences of Pf4 integration into LESB58 and PA14 genome.
