# Supplementary material for: Filamentous Pseudomonas Phage Pf4 in the Context of Therapy-Inducibility, Infectivity, Lysogenic Conversion, and Potential Application
Source: Viruses. 2022 Jun 10;14(6):1261. doi: 10.3390/v14061261 (PMC9228429; doi:10.3390/v14061261)
Supplement: Supplementary file 1 [file viruses-14-01261-s001.zip › viruses-1738559-SM-update.pdf]

Table S1. Primers used in the study.

Purpose	Label of Primer	Gene function	Sequence (5' -> 3')	Expected product (bp)	Reference
RT-qPCR	RpoD-F	Sigma factor 70	CGT TGA TCC CCA TGT CGT T	155	This study.
	RpoD -R		GCA ACA GCA ATC TCG TCT GA		
	ProC-F	Pyrroline-5-carboxylate reductase	CAG GCC GGG CAG TTG CTG TC	180	Salvi et al., 2003; [79]
	ProC -R		GGT CAG GCG CGA GGC TGT CT		
	Pf4CoaB-F	Major capsid protein (CoaB)	GCA ACG CAT CGC CAA GTT	113	This study.
	Pf4CoaB -R		CGC TGG TGT CGA TCA CCC		
	Pf4Zot-F	Zonula occludens toxin (Zot)	GAT GTT CGG CGT GGT GAG	180	This study.
	Pf4Zot-R		ATC TTC GAC GAA ACC CAA CTG		
	Pf4CoaA-F	Minor capsid protein (CoaA)	GTG CCG ATT CCT ACA CCT TC	135	This study.
	Pf4CoaA-R		CCT TGA CGC AGG TAG TTC CC		
	PfLES58CoaA-F	Minor capsid protein (CoaA)	ATC GAA CTT GGA AGC GAG GA	110	This study.
	PfLES58CoaA-R		GCT CCT TCG ACA AGA CCA TC		
	Pf5CoaA-F	Minor capsid protein (CoaA)	CGT GTT GAT GAC GGA CGG TA	134	This study.
	Pf5CoaA-R		GAG GAC AGC CAG GGT CAT TC		
	Pf4RF-F	Replicative form (RF)	CTT GGC AGG GTG ATT TGG A	95	This study.
	Pf4RF-R		AGG AAC GCT TCA AAA CCC TA		
	PfLES58RF-F	Replicative form (RF)	ACG GCT CTG CAC TTC TAC G	120	This study.
	PfLES58RF-R		CGA CAG TTC TTC GAC ACT TGC		
	Pf5RF-F	Replicative form (RF)	GCC TAG CGT TGA CCA GTT A	78	This study.
	Pf5RF-R		CTT TGC CGA TTT GTG CGT A		
PCR/RFLP	IntF4-F	Integrase (Int)	TCG AAT TCC GCT TCC ATC AC	1001	Knezevic et al., 2015; [14]
	IntF4-R		CCT GAT GCT TGG TCA GGT ACG		
	Pf4RFc-F	Replicative form (RF)	AGC AGC GCG ATG AAG CAA T	865	

Pf4RFc -R		TAG AGG CCA TTT GTG ACT GGA		Rice et al., 2009; [13]
Pf4/LESB58-F	Pf4 integration site in LESB58 genome	CAA TGG TCG TCA CGC AGA AC	943	This study.
Pf4/LESB58-R		CCG CTC AAC CCG ATC TAC		
Pf4/PA14-F	Pf4 integration site in PA14 genome	CAA TGG TCG TCA CGC AGA AC	943	This study.
Pf4/PA14-R		CCG CTC AAC CCG ATC TAC		

Table S2. Sequences of Pf4 integration into LESB58 and PA14 genome

>LESB58+Pf4
CGGACGGTGTTCCTGCCTATGTGGTTGAGTCATATGCTGAGCGGAAGCAGCGCGATGAAGCAATTG
CGCTGGTGAAGTTGCTTGCGATTGGCTCCCGCCAGTACGCAGAAGGCAAGCATCGCTCTGTTGATGA
TTTGAAAGCTCGCCTTTCCAGGAGGTTCGCTCAGCCAGAATAAGGAGGTTTAATGTCCCCGGTCGTC
ATTCGTTTTACTGATACCGCAGAGCAAAGCATCGAAGACCAAGTCCACCACTTGGCTCCATTCCAAG
GTGAACAGGCTGCACTCCAGTCAGTACTGAGCCTTTTGGATGAGATTGAAGAGAAGATTTCACTTGC
ACCTAAAGGTTACCCAGTCAGCCAGCAGGCGAGTCTTCTGGGGGTGCTGAGCTATCGCGAGCTTAA
TACCGGCCCCTATCGTGTTTTTTACGAATTCCACGAAGAGCAAGGCGAGGTGGCAGTGATCTTGGTT
TTGCGACAGAAGCAGAGCGTTGAGCAGCAATTGATCCGCTACTGCTTGGTGGGGCCAATCGAGTGA
TGGCTTTCTACTCCTGAGCATGTAGCGCTGAATGCGCCTCGACACTTCTTCGACACCTTTCCTTCCCC
CAAAAAGCAAAGCCCCCGAAACGCTAGGCATTTCAGGGGCTTGGCAGGGTGATTTGGAGCGGGCG
AAGGGAATCGAACCCTCGTCATGAGCTTGGGAAGCTCAGGTAATGCCATTATACGACGCCCGCTCG
GACGGCTTTTGCGGCCAGGGCGCCTTTTTACCAGATGCGCGGCGGCAGGTGAAGCCCGGGGCGGGG
GTTTTTGTTGATTTCGCTGGGTTTTTCCGCCAGGGGGAGCGGGGCTCCCCGTGGCGGTGGCGGTTAG
CTGGCGAGGGCGGCGAGGGGGACGCTGGCGCCCGTTGGGCTGGGGGCGTATG

>PA14+Pf4
GGGTCTGCCTATGTGGTTGAGTCATATGCTGAGCGGAAGCAGCGCGATGAAGCAATTGCGCTGGTG
AAGTTGCTTGCGATTGGCTCCCGCCAGTACGCAGAAGGCAAGCATCGCTCTGTTGATGATTTGAAAG
CTCGCCTTTCCAGGAGGTTCGCTCAGCCAGAATAAGGAGGTTTAATGTCCCCGGTCGTCATTCGTTT
TACTGATACCGCAGAGCAAAGCATCGAAGACCAAGTCCACCACTTGGCTCCATTCCAAGGTGAACA
GGCTGCACTCCAGTCAGTACTGAGCCTTTTGGATGAGATTGAAGAGAAGATTTCACTTGCACCTAAA
GGTTACCCAGTCAGCCAGCAGGCGAGTCTTCTGGGGGTGCTGAGCTATCGCGAGCTTAATACCGGC
CCCTATCGTGTTTTTTACGAATTCCACGAAGAGCAAGGCGAGGTGGCAGTGATCTTGGTTTTGCGAC
AGAAGCAGAGCGTTGAGCAGCAATTGATCCGCTACTGCTTGGTGGGGCCAATCGAGTGATGGCTTT
CTACTCCTGAGCATGTAGCGCTGAATGCGCCTCGACACTTCTTCGACACCTTTCCTTCCCCCAAAAA
GCAAAGCCCCCGAAACGCTAGGCATTTCAGGGGCTTGGCAGGGTGATTTGGAGCGGGCGAAGGGA
ATCGAACCCTCGTCATGAGCTTGGGAAGCTCAGGTAATGCCATTATACGACGCCCGCTCGGACGGC
TTTTGCGGCCAGGGCGCCTTTTTACCAGATGCGCGGCGGCAGGTGAAGCCCGGGGCGGGGTTTTTGT
TGATTTCGCTGGGTTTTTCCGCCAGGGGGAGCGGGGCTCCCCGTGGCGGTGGCGGTTAGCTGGCGA
GGGCGGCGAGGGGACGCTGGCGCCGTTGGG

Pf4	tRNA-Gly	Bacterial genome
